# Investigating the Grammatical and Pragmatic Origins of Wh-Questions in Children with Autism Spectrum Disorders

**DOI:** 10.3389/fpsyg.2017.00319

**Published:** 2017-03-10

**Authors:** Manya Jyotishi, Deborah Fein, Letitia Naigles

**Affiliations:** Department of Psychological Sciences, University of ConnecticutStorrs, CT, USA

**Keywords:** wh-questions, language, comprehension, grammar, social-pragmatics, SVO word order

## Abstract

Compared to typically developing children, children with autism (ASD) show delayed production of wh-questions. It is currently controversial the degree to which such deficits derive from social-pragmatic requirements and/or because these are complex grammatical structures. The current study employed the intermodal preferential looking (IPL) paradigm, which reduces social-pragmatic demands. The IPL paradigm can help distinguish these proposals, as successful comprehension promotes the “pragmatics-origins” argument whereas comprehension difficulties would implicate a “grammatical-origins” argument. Additionally, we tested both the linguistic and social explanations by assessing the contributions of children's early grammatical knowledge (i.e., SVO word order) and their social-pragmatic scores on the Vineland to their later wh-question comprehension. Fourteen children with ASD and 17 TD children, matched on language level, were visited in their homes at 4-month intervals. Comprehension of wh-questions and SVO word order were tested via IPL: the wh-question video showed a costumed horse and bird serving as agents or patients of familiar transitive actions. During the test trials, they were displayed side by side with directing audios (e.g., “What did the horse tickle?”, “What hugged the bird?”, “Where is the horse/bird?”). Children's eye movements were coded offline; the DV was their percent looking to the named item during test. To show comprehension, children should look longer at the named item during a where-question than during a subject-wh or object-wh question. Results indicated that TD children comprehended both subject and object wh-questions at 32 months of age. Comprehension of object-wh questions emerged *chronologically* later in children with ASD compared to their TD peers, but at similar levels of *language*. Moreover, performance on word order and social-pragmatic scores independently predicted both groups' later performance on wh-question comprehension. Our findings indicate that both grammar and social-pragmatics are implicated in the comprehension of wh-questions. The “grammatical-origins” argument is supported because the ASD group did not reveal earlier and stable comprehension of wh-questions; furthermore, their performance on SVO word order predicted their later success in linguistic processing of wh-questions. The “pragmatic-origins” argument is also supported because children's earlier socialization and communication scores strongly predicted their successful performance on wh-question comprehension.

## Introduction

According to the DSM-V (American Psychiatric Association, 2013), autism spectrum disorder (ASD) is characterized as a developmental disorder with persistent deficits in social interaction and social communication, and with restricted and repetitive patterns of behaviors. Researchers have also proposed that some aspects of language development are different and/or delayed in this population compared to typically developing (TD) children (Rutter, 1978; Charman et al., 2003; Mitchell et al., 2006). It is generally acknowledged that children with ASD have underlying pragmatic deficits attributable to their social-communicative impairment (Kjelgaard and Tager-Flusberg, 2001; Tager-Flusberg et al., 2005; Naigles and Chin, 2015); however, the extent to which a grammatical deficit is also involved continues to be a matter of controversy (Tager-Flusberg, 1994; Eigsti et al., 2007; Eigsti and Bennetto, 2009; Naigles and Chin, 2015; Naigles and Fein, 2017). One way to investigate the extent of social-pragmatic difficulties and grammatical deficits in ASD is to examine their acquisition of wh-questions.

The acquisition of wh-questions seems challenging for children with ASD, as prior research has shown delays in both production and comprehension (Tager-Flusberg, 1994; Goodwin et al., 2012). Some researchers have argued that children with ASD have particular difficulties with wh-questions because these are complex grammatical structures (Eigsti et al., 2007) while others have proposed that their impairments are more related to pragmatics (Tager-Flusberg, 1994). However, most studies that have tested wh-questions in this population have involved spontaneous production, which relies heavily on social-pragmatics knowledge; e.g., knowing how to use these questions in the appropriate contexts. We examine whether there is also a grammatical deficit by investigating whether children with ASD comprehend subject-wh and object wh-questions during the same developmental period as their TD peers, using a paradigm that minimizes social-pragmatic demands. If wh-question difficulties have grammatical origins in these children, then these would also be implicated in their *understanding* of wh-questions. Moreover, to further explore the grammatical-origins argument, we examined the relationships between earlier grammatical and social competences and later wh-question comprehension.

Wh-question acquisition is interesting because these constructions require both grammatical and pragmatic knowledge. A wh-question is a question that contains a wh-word (*what, where, when, why, how*), usually occurring in the beginning of the sentence (in English). Syntactically, these wh-words stand for information that is missing in the sentence. Wh-questions probe for missing arguments (e.g., “What did Mary buy?”) or adjuncts (e.g., “Why did she buy that?”). Furthermore, argument wh-questions can ask for the grammatical subject of a sentence (e.g., (1) Who __ likes Mary?) or the grammatical object of the sentence (e.g., (2) Who does Mary like __?). Notice that both subject and object wh-questions involve wh-movement from the original argument location; however, the movement for subject wh-questions does not change the canonical word order of English sentences (SVO; see (1) above), whereas the movement for object wh-questions changes the word order of the sentence to OSV [see (2) above; Radford, 1988; Ambridge and Lieven, 2011].

Pragmatically, wh-questions serve several communicative functions. Wh-questions ask for information, which is unknown but desired by the speaker and is assumed to be known by the addressee. Moreover, the speaker needs to have knowledge about when such questions are proper to use in a discourse/conversational setting (Searle, 1969). Specifically, children can ask questions to seek new factual information from the listener about social or public information or elaborate about shared information between the speaker and listener; their questions can ask for clarifications or repetitions about the conversation, and they can reflect the speaker's knowledge, such as, rhetorical questions, or didactic questions (Sinclair and Van Gessel, 1990; Freed, 1994). Some wh-questions can ask for information about motives, intentions, or mental states of others (Gauvain et al., 2013; e.g., Where do you think the ball went?), whereas other types of wh-questions target purely physical objects, locations and events, such as, “Where's the bear?” or “What are you cooking?” These latter questions do not require mentalization to interpret the correct answer but nonetheless have underlying pragmatic functions like information seeking about objects and events, probing about shared events and experiences, and providing a conversational focus during play.

Wh-questions are acquired by TD children during the preschool years, with comprehension of subject and object wh-questions attested between 1 and 2 years of age (Seidl et al., 2003; Goodwin et al., 2012; Gagliardi et al., 2016), and production of the same forms observed by 24–30 months (Tyack and Ingram, 1977; Bloom et al., 1982; Stromswold, 1995). Two- to three-year old children first use these questions for information-seeking purposes, such as, “Where is the washcloth?” or “What are they drinking?” (Tyack and Ingram, 1977; Bloom et al., 1982; Goodwin et al., 2015), and soon also use the questions for conversational purposes like initiating or maintaining conversations, such as, “How are you?” or “What's that?” Some questions also serve a directive function, such as, “Why don't we read this one?” (James and Seebach, 1982).

Production of wh-questions also emerges during the preschool years for verbal children with ASD, but seems to be both delayed and sparse. For example, during structured and free play sessions, verbal children with ASD were observed to request less information compared to their TD peers and used fewer wh-questions during naturalistic (i.e., unprompted) interactions (Wetherby and Prutting, 1984; Tager-Flusberg, 1994; Eigsti et al., 2007; Goodwin et al., 2012). Early hypotheses concerning the origins of this “wh-question deficit” have focused on the social/pragmatic impairments of children with ASD, arguing that the children were less interested in soliciting information from others, and so had fewer reasons to ask the questions (Rutter, 1978; Tager-Flusberg, 1994). Children with ASD might also ask fewer wh-questions because of their impaired understanding that others can have knowledge that would inform the purpose of their questions. Tager-Flusberg's (1994) analysis of the spontaneous speech of six boys with ASD supported this hypothesis, because while the boys increased in their production of well-formed wh-questions over time—especially in using auxiliary verbs and inversion—at rates similar to language-matched peers, their overall frequency of wh-question usage remained sparse (9.3 per 1000 utterances in the ASD group vs. 28.2 per 1000 utterances in the language-matched peers). More qualitatively, the children with ASD's usage of wh-questions in conversations was more restricted, i.e., they produced fewer information-seeking questions about objects, events or psychological states, and did not seem to manifest the conversational functions of agreement and clarification to regulate verbal interactions. Children with ASD also rarely asked conversational openers or social routine questions like, “How are you?” Thus, children with ASD did not seem impaired in their syntactic acquisition of wh-question forms, as shown by their growth in well-formed questions, but their usage of these questions was clearly impoverished.

The pragmatic-origins hypothesis has also been supported by Goodwin et al. (2012), who examined wh-question comprehension in English-speaking children with ASD using intermodal preferential looking (IPL). IPL has the potential to provide a more accurate assessment of linguistic knowledge in very young children, because it involves little to no social, motor or speech demands: children simply watch two videos while hearing a central audio that matches only one of the videos. The children's eye movements are recorded; the assumption is that if they understand the audio, they will look longer at the matching video (Golinkoff et al., 1987, 2013). IPL thus reduces the social-pragmatic constraints for the use of wh-questions; children are not asked to answer any questions, nor are they expected to produce any. Goodwin et al. (2012) showed a wh-question video to TD children and children with ASD at four visits during a longitudinal study. The video presented familiar items—an apple, a flower, keys, and a book—engaged in hitting events (i.e., an apple hitting a flower, keys hitting a book; adapted from Seidl et al., 2003). Following these familiarization trials, the children saw three test trials that asked object-wh, subject-wh, and “where” questions while the pairs of items were displayed simultaneously, side by side. The TD children demonstrated reliable understanding of wh-questions at 28 months of age, at the first visit when they were shown the videos. The children with ASD showed reliable comprehension only at 54 months of age, at the 4th visit when they had seen the videos. While their comprehension was delayed relative to the TD group in terms of their chronological age, the overall language level of the ASD group at 54 months was not different from the language level of the TD children at 28 months; therefore, Goodwin et al. (2012) suggested that comprehension of wh-questions was achieved at similar language levels in both groups. Minimizing the pragmatic demands of wh-question use via IPL yielded positive findings of wh-question *knowledge*, thus supporting the claim that sparse wh-question *usage* in children with ASD is a result of their social/pragmatic impairments. The findings of Durrleman et al. (2016) are also consistent with this hypothesis. These researchers tested school-age French children with ASD on their comprehension of both simple and complex wh-questions, and reported that, while the children performed above chance, their scores were significantly lower than those of TD children matched on non-verbal abilities.

However, not all research is consistent with the pragmatic origins hypothesis. Two recent studies of the spontaneous speech of children with ASD have indicated that their wh-question development was tightly linked to their overall grammatical development. Eigsti et al. (2007) compared five-year-old children with ASD to TD children matched on non-verbal IQ and receptive vocabulary. Not surprisingly, the children with ASD used fewer and less complex wh-questions than the TD children; however, they also had smaller mean length of utterance (MLUs), indicating that their syntactic development was delayed relative to their vocabulary levels. Moreover, their wh-question complexity patterned with their MLU rather than their vocabulary. Tek et al. (2014) followed two subgroups of children with ASD across 2 years, and found that the high-verbal children with ASD, who were matched on MLU with TD children, showed increases in their complexity of wh-question use (i.e., progressing from routine questions to wh-questions with verbs, and then to wh-questions with both a main and auxiliary verb, etc.) that paralleled the increases in their MLU and in the wh-question use of the TD group. In contrast, the low-verbal children with ASD showed flatter slopes in their individual growth curves. In sum, these researchers have found wh-question use in children with ASD to be commensurate with their overall grammatical levels, suggesting that observed deficits are due to grammatical difficulties rather than pragmatic ones.

In the current study, we revisit this debate concerning the grammatical vs. pragmatics origins of the wh-question deficit in two ways. First, we conducted a replication and extension of Goodwin et al.'s (2012) study, altering the stimuli with the goal of making them easier. Second, we investigated possible precursors to wh-question comprehension, under the hypothesis that if the wh-question deficit has a grammatical origin, then early grammatical competence will predict later wh-question comprehension; in contrast, if the wh-question deficit has a pragmatics origin, then early social competence will predict later wh-question comprehension. We motivate each of these innovations below.

Goodwin et al. (2012) reported that the children with ASD achieved wh-question comprehension at the visit when their general language levels were on a par with those of the TD children, at the first visit when they (the TD children) demonstrated wh-question comprehension. Following Seidl et al. (2003) and Gagliardi et al. (2016), who reported successful wh-question comprehension in TD children as young as 20 months of age, it is possible that the TD children in Goodwin et al. (2012) would have shown comprehension at lower language levels; however, they were not shown this video at earlier visits. The children with ASD in Goodwin et al. *were* tested on wh-question comprehension when their language levels were at age-equivalents of 20 months, but they did not show comprehension at this earlier point. We conjecture, though, that several aspects of Goodwin et al.'s (2012) stimuli were less than ideal. First, both events involved the verb *hit*, which we have found is not common for children with ASD. That is, even by 54 months of age, only 53% of the children with ASD had produced the verb “hit,” according to parental report. If “hit,” and hitting events, are unfamiliar to young children with ASD, they might not have been able to process the wh-questions efficiently during the 4-s test trials. In contrast, all TD children in the study had produced this verb at 32 months of age—and most showed successful wh-question comprehension as well. Furthermore, the hitting events themselves were non-prototypical transitive events; that is, they involved the action of an inanimate agent on an inanimate patient. Prototypical transitive events involve animate agents (Slobin, 1982), as do prototypical wh-questions (Tyack and Ingram, 1977), and the wh-questions produced by children with ASD generally follow this pattern as well (Tager-Flusberg, 1994; Tek et al., 2014). The presentation of inanimate agents might have caused additional confusion. In sum, it is possible that earlier comprehension of wh-questions in these children with ASD was not demonstrated due to these challenging stimuli, and the current study introduces several changes which were hypothesized to facilitate the interpretation of the events and so the comprehension of wh-questions referring to those events. Evidence of earlier comprehension would support the “pragmatic origins” hypothesis.

A second way to examine the origins of wh-question acquisition, and of the deficit observed in the productions of children with ASD, is to investigate the extent to which earlier grammatical and/or pragmatic factors are precursors or predictors of successful wh-question comprehension. Grammatically, a pre-requisite to understanding subject- and object-questions might lie in children's understanding of basic declarative sentences consisting of a subject, a verb, and an object, known as canonical English SVO word order. For example, in order to engage in wh-movement, children should have systematically understood the SVO sentence structure (3) and one-to-one matched the structure of the frame with the wh-question (4) to help them guide to the correct referent (either the subject or object) of the action. 
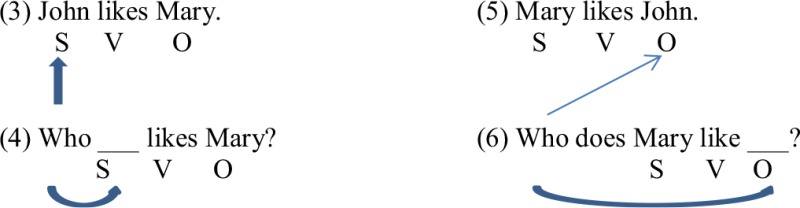


In the above example, if children have understood the subject-verb-object sentence structure from hearing the sentence “John likes Mary,” then when they hear a subject-wh-question like, “Who __ likes Mary?” children should be able to structurally map this transitive construction to the gap in the subject position of the question, “Who ___ likes Mary?” Moreover, if children understand that the SVO sentence structure is a transitive frame with a subject (a “*liker*”) and a verb (“*like*”) that requires a direct object (a “*like*”) this knowledge can enable them to map the wh-word movement back to its gap in the object position. Therefore, we propose to investigate how children's prior grammatical knowledge of SVO word order contributes to their later wh-question comprehension. Research with TD children has begun to demonstrate that early sentence processing skills predict later syntactic performance (Newman et al., 2006; Kidd and Arciuli, 2016); in addition, one recent study has found predictive relations between children with ASD's processing of sentences and their later sentence comprehension (Naigles et al., 2011). In that study, children with ASD were taught novel verbs in transitive sentences via the IPL paradigm and then asked whether the verbs mapped onto causative or non-causative actions; i.e., syntactic bootstrapping (Naigles, 1990). The children were generally successful; moreover, after controlling for their vocabulary size, those who were faster processors of SVO word order (i.e., showing a shorter latency to look at the match scene) 8 months earlier were better able to use the SVO frames to make predictions about new verb meaning (children's longer looking time toward the matching scene during the test trials compared to baseline trials). In the current study, we investigate the extent to which children's comprehension of wh-questions is predicted by their earlier comprehension of declarative SVO sentences.

Pragmatic prerequisites to children's acquisition of wh-questions *per se* are less well-defined; however, pragmatic and social precursors to language development in general are well-attested, and include such factors as joint attention, gesture, and turn-taking (Clark, 2015; Tomasello, 2015). These behaviors are known to be consistently impaired in children with ASD (Tager-Flusberg et al., 2005), and variability in early manifestations of these pragmatic abilities has been found to predict variability in later measures of language, both general (Mundy et al., 1990; Luyster et al., 2008) and specifically grammatical (Rollins and Snow, 1998; Naigles et al., 2016). In the current study, we directly investigate the contribution of social and pragmatic factors to wh-question development and understanding, and hypothesize that children who are more attuned to their social and communicative milieu might acquire wh-questions earlier, because by attending well to their functions (e.g., asking for information), they may also become focused sufficiently on their forms.

In the current study, we used IPL to assess wh-question comprehension in TD preschoolers and preschoolers with ASD. We created new videos that included animate characters, i.e., a costumed horse and a bird, as well as new actions and verbs, such as *tickle, wash, hug*, and *ride*, which have been reported to be understood by children with ASD at 2.5 years of age (Swensen et al., 2007). Our first hypothesis was that finding earlier or equivalent comprehension with these videos, compared to those of Goodwin et al. (2012), would support a pragmatic origin for the “wh-question deficit” in children with ASD. That is, minimizing pragmatic demands, coupled with more familiar stimuli, should illuminate intact grammatical knowledge. In contrast, later or weaker wh-question comprehension with the new videos would be consistent with a grammatical origin.

We also examined the relationships between children's early standardized test measures, socialization measures, and word order comprehension, and their later wh-question comprehension to investigate the degree to which earlier general language measures or social measures are related to later comprehension. In terms of grammatical competence, early grammatical knowledge of word order may serve in either general or specific ways as a foundation for later acquisition of wh-questions. For example, in general, if a child has difficulties acquiring word order at an early age then these same difficulties could influence their ability to learn grammar in later years. Specific links between early acquisition of word order and wh-question comprehension might involve the fact that without understanding that SVO is the canonical word order in English, the function of the wh-word, i.e., that it stands for a missing NP, might be opaque. Our study was not designed to distinguish between these possibilities; instead, we investigate whether early grammatical competence is associated with later performance on wh-questions, which would strengthen the argument of a grammatical deficit in wh-questions in children with ASD. We also investigate whether early (rather than concurrent) social competence is related to subsequent wh-question comprehension, on the rationale that children need to be socially aware to understand the point of wh-questions and the reasons for asking them. For example, one Vineland question asks, “Answers when familiar adults make small talk (for example, if asked, “How are you?” says, “I'm fine”; if told, “You look nice”,” says, “Thank you”; etc.). Thus, if early socialization measures are associated with later wh-question comprehension, then this will support the pragmatics-origins argument.

## Materials and methods

### Participants

Fourteen children with ASD and 17 TD children participated in this longitudinal study. All were monolingual English learners. One child with ASD participated in the overall project, but was not included in the final analyses of this study because he did not provide sufficient data during the wh-question task for more than half of the visits. One child in the TD group was omitted from the IPL analyses at visit 6 because she had missing data at this visit. We recruited participants in the ASD group by contacting facilities that offer Applied Behavioral Analysis (ABA; Lovaas, 1987); we restricted the sample to children receiving ABA to ensure some consistency in the interventions being received. Moreover, ABA is the most common intervention offered in our geographic area (northeastern U.S.). These service providers distributed information about the study to parents of children who had been diagnosed within the last 6 months and had just begun ABA training. Interested parents then contacted us and were interviewed via telephone to verify their child's diagnosis and eligibility for the study. All parents signed consent forms prior to participating.

The participants in the ASD group included seven White males, two Asian males, and one African American male. There were two White females, one Asian female, and one African American female. This sample of children somewhat reflects the prevalence of ASD in the general population; we made significant efforts to recruit non-Caucasian families. All children were from lower-to upper-middle-class families living in the Northeastern United States. At the first visit, the children with ASD ranged in age from 18 to 42 months (*M* = 32.93, *SD* = 7.28) and their MLU, a measure of sentence complexity, ranged from 0 to 3.13 (*M* = 1.26, *SD* = 0.67). To be included in the study, the children with ASD had to be receiving at least 20 hours of ABA intervention weekly. Because it is difficult to distinguish between ASD and pervasive developmental disorder—not otherwise specified (PDD-NOS), we accepted participants with either diagnosis, which was then verified by the Autism Diagnostic Observation Schedule (ADOS; Lord et al., 2000). The ADOS and other test scores are provided in Table 1.

**Table 1:** **Standardized test data for Typically Developing (TD) and Autism Spectrum Disorder (ASD) groups at their first and final visits (***M, SD***)**.

	**TD**	**ASD**	***t***	***p*****-values**
**VISIT 1**				
Gender	13 boys, 4 girls	10 boys, 4 girls		
ADOS	1.47 (1.66)	14.50 (3.70)	−12.21	<0.001
Range^a^	0–5	7–21		
CARS	16.21 (1.96)	37.96 (6.10)	−12.81	<0.001
Range^b^	15–22.5	31–52		
**CDI (infant version)^c^**				
Word production	123.59 (108.15)	66.21 (113.60)	1.44	0.161
**Mullen raw scores**				
Visual reception	25.88 (3.46)	27.57 (5.37)	−1.06	0.299
Fine motor	22.59 (2.60)	25.07 (4.20)	−2.02	0.053
Receptive language	22.76 (3.87)	19.64 (10.37)	1.07	0.302
Expressive language	20.35 (5.70)	16.29 (6.64)	1.84	0.077
**Mullen T-scores**				
Visual reception	59.35 (11.37)	36.57 (15.12)	4.79	<0.001
Fine motor	53.41 (10.95)	33.43 (16.81)	3.99	<0.001
Receptive language	55.53 (13.26)	33.79 (19.62)	3.67	0.001
Expressive language	51.71 (15.05)	26.50 (8.86)	5.52	<0.001
**Vineland standard scores**				
Communication	105.12 (9.87)	72.07 (15.45)	7.22	<0.001
Daily Living	103.76 (9.46)	79.50 (15.05)	5.47	<0.001
Socialization	101.71 (6.08)	73.07 (8.53)	10.90	<0.001
Motor	98.06 (6.79)	87.64 (14.85)	2.42	0.026
**VISIT 3**				
CDI (toddler version)	456.06 (136.69)	178.75 (169.96)	4.79	<0.001
**VISIT 4**				
ROWPVT standard scores	115.81 (14.90)	86.35 (24.82)	3.87	0.001
EOWPVT standard scores	104.88 (12.59)	80.17 (27.83)	2.88	0.012
**VISIT 5**				
ROWPVT standard scores	120.23 (13.06)	91.78 (23.26)	4.07	0.001
EOWPVT standard scores	111.70 (16.39)	79.61 (26.56)	3.83	0.001
**VISIT 6**				
MLU	2.76 (0.54)	1.97 (0.90)	2.81	0.011
ROWPVT standard scores	125.56 (11.86)	97.07 (23.95)	4.04	0.001
EOWPVT standard scores	114.12 (16.46)	77.00 (34.98)	3.64	0.002
**Mullen raw scores**				
Visual reception	43.56 (4.02)	40.00 (7.67)	1.56	0.135
Fine motor	38.56 (5.11)	33.93 (7.11)	2.07^*^	0.048
Receptive language	40.31 (4.88)	34.21 (9.35)	2.19^*^	0.041
**Mullen T-scores**				
Visual reception	63.81 (11.32)	40.50 (18.97)	4.02^*^	0.001
Fine motor	59.50 (16.32)	31.86 (17.85)	4.43^*^	<0.001
Receptive language	63.13 (10.90)	37.21 (20.27)	4.27^*^	<0.001
Expressive language	59.88 (10.73)	35.00 (22.48)	3.78	0.001

* *p < 0.05*.

a *Autism spectrum = 7+; autism = 12+*.

b *CARS range = 15–60; Autism spectrum = 30+; autism = 36+*.

c *Number of words produced out of 396*.

*ADOS, Autism Diagnostic Observation Schedule; CARS, Childhood Autism Rating Scale; CDI, Communication Development Inventory. ROWPVT, Receptive One-Word Picture Vocabulary Test. EOWPVT, Expressive One-Word Picture Vocabulary Test*.

The TD group was recruited via birth announcements from local newspapers. The TD group included 13 White males, three White females and one Asian female from middle- to upper-middle-class families living in Connecticut. These demographics closely resembled those of the ASD group. Rather than matching the TD group to the ASD group on age, we chose to match them on level of language development. Therefore, we began testing TD children at ~20 months of age (*M* = 19.74, *SD* = 1.25) with MLU ranging from 1.02 to 1.86 (*M* = 1.36, *SD* = 0.25) at visit 1, when their language abilities were most similar to those of the ASD group at visit 1 (see Table 1).

### Materials

#### Standardized tests

The ADOS (Lord et al., 2000) was administered to assess ASD status. We also administered the Vineland Adaptive Behavior Scales, 2nd Edition (Vineland II; Sparrow et al., 2005) to evaluate children's communication, socialization, daily living skills, and motor skills, which yielded standard scores based on mothers' reports. The communication domain of the Vineland consisted of some items related to language competence, such as, “Uses present tense verbs ending in *ing* (for example, “Is singing”; “Is playing”; etc.),” and other items that were more related to pragmatics, such as, “Understands sayings that are not meant to be taken word for word (for example, “Button your lip”; “Hit the road”; etc.)” or “Asks questions by changing inflection of words or simple phrases (for example, “Mine?”; “Me go?”; etc.)”; grammar is not important. The socialization domain consisted of items like, “Makes or tries to make social contact (for example, smiles, makes noises, etc.)” or “Answers when familiar adults make small talk (for example, if asked, “How are you?” says, “I'm fine”; if told, “You look nice,” says, “Thank you”; etc)”. In the ASD literature, the Vineland scale has been found to be strongly correlated with join attention skills (Toth et al., 2006; Poon et al., 2012) and ADOS scores (Klin et al., 2007; Paul et al., 2014); it is frequently used as a measure of social competence in special populations like ADHD and ASD (Oswald and Ollendick, 1989; Charman et al., 2001, respectively). In our study, an average of the communication and socialization scores was used as a measure of social competence.

The Mullen Scales of Early Learning (1994) were administered to measure the development in the areas of visual perception, fine motor skills, receptive language, expressive language, and gross motor skills (Mullen, 1994). Finally, the MacArthur Communicative Developmental Inventory (CDI; Fenson et al., 1994) provided a measure of the child's production vocabulary, via parental report. The infant version of the CDI was used at visit 1. The Receptive One-Word Picture Vocabulary Test, 4th edition (ROWPVT-4; Martin and Brownell, 2010b) and Expressive One-Word Picture Vocabulary Tests, 4th edition (EOWPVT-4; Martin and Brownell, 2010a) were administered at all visits to evaluate the children's receptive and expressive vocabulary skills, respectively.

#### IPL setup

The IPL paradigm (Golinkoff et al., 1987; Naigles and Tovar, 2012) involves showing children two videos side by side, while playing child–directed speech from a central speaker that corresponds to only one of the videos. The child's direction and duration of gaze are recorded and coded for indications of his/her understanding. An Apple Powerbook was used to project the stimuli onto a portable 63” × 84” screen, via an LCD projector. The computer was connected to an external speaker, which was placed out of sight behind the screen. A digital camcorder for filming the child's face was placed on a small tripod in front of the screen, just below the center.

### IPL stimuli

#### Wh-question

The wh-question video was adapted from Goodwin et al. (2012), with two major changes. First, the animate characters of a costumed horse and bird served as agents and patients. Second, these characters engaged in four familiar live-action transitive events: washing, tickling, riding and hugging. The verbs describing these events were all attested in the vocabularies (i.e., CDIs) of both groups by visit 4. The horse appeared as the agent for the tickle and ride events, and the bird appeared as the agent for the wash and hug events. After each transitive event, the horse and bird appeared side by side and the audio asked a wh-object or wh-subject question. In total, each child was asked four object-wh-questions, four subject-wh-questions, and at the end of the video, two where-questions. In the videos, the side of the matching scene was counterbalanced both within (i.e., the matching side varied from left to right in an XYYXXY pattern) and between (i.e., for half of the children the first match was on the left and for the other half, the first match was on the right) participants (see Table 2 for the layout).

**Table 2:** **Sample layout of the Wh-question video**.

**Trial type**	**Audio**	**Video 1**	**Video 2**
2 Control-baseline	They're on both screens!	Bird	Horse
4 Familiarization	Look at this!	Horse tickles bird	Black
6 Familiarization	See this?	Black	Horse tickles bird
8 Test^a^	What did the horse tickle __?	Bird	Horse
28	(Block repeats with *wash/hug/ride*) Isn't this fun?		
30 Familiarization	Look at this!	Bird hugging horse	Black
32 Familiarization	See this?	Black	Bird hugging horse
34 Test^b^	What ___hugged the horse? (Block repeats with *ride/tickle/wash*)	Bird	Horse
54	Isn't this fun?	Screensaver	Screensaver
56 Where-test^c^	Find the horse!	Bird	Horse
58 Where-Test^c^	Find the bird!	Bird	Horse

a Object-wh-questions = What did the horse tickle?; What did the bird wash?; What did the bird hug?; What did the horse ride?

b Subject-wh-questions = What hugged the horse?; What rode the bird?; What tickled the bird?; What washed the horse?

c Where is the horse?; Where is the bird?

#### Word order (Candan et al., 2012)

The layout for the word order video is presented in Table 3. The pretest trials (labeled “P” in the table) introduced and labeled the costumed horse and bird. Trials 1–2 presented a familiar action with agent A and patient B on one side (e.g., the bird pushing the horse), and then with agent B and patient A on the other side (e.g., the horse pushing the bird). During these trials, the action was labeled in a neutral frame (e.g., “Pushing!”). In Trial 3 (the control-for-salience trial), both renditions of the action were presented simultaneously and the audio was the same as in trials 1 and 2; this provided a baseline measure of stimulus salience. Trial 4 was the test trial, in which the verb was placed in a sentence such that only one of the two renditions matched. This trial thus examined whether the child understood the difference between “A verbs B” (e.g., “the bird is pushing the horse”) and “B verbs A” (e.g., “the horse is pushing the bird”). A total of six familiar verbs and actions were introduced and then tested for word order understanding. These were *push, tickle, pull, wash, hug*, and *ride*. The same characters were used for each action; the horse was the agent for half of the matching actions and the bird was the agent for the others.

**Table 3:** **Sample layout of the word order video**.

**Video 1**	**Audio**	**Video 2**
P^a^ Horse waves	Look, a horse! See, the horse!	Blank
P Blank	Look a bird! See, the bird!	Bird waves
P Horse waves	We see both!	Bird waves
P Horse waves	Look at the horse!	Bird waves
P Horse waves	Look at the bird!	Bird waves
1 Blank	Look, pushing! See, pushing!	Bird pushes horse
2 Horse pushes bird	Look, pushing! Wow, pushing!	Blank
3 Horse pushes bird	They are on both screens!	Bird pushes horse
4 Horse pushes bird	Look, the bird is pushing the horse! (Block repeats with tickle/pull/wash/ hug/ride)	Bird pushes horse

a *P indicates the pretest trials*.

### Procedure

The children were visited in their homes, at 4-month intervals for a total of six visits. The visits began with one experimenter administering standardized tests, while another experimenter prepared the IPL setup. Next, the child sat ~3 ft in front of the screen and camcorder and watched three IPL videos. The word order video was shown at visits 1 and 2; the wh-question video was shown at visits 3 through 6, and was always the second or third video in the series. Breaks were allowed as needed between videos. After viewing the videos, the mother and child participated in a 30-min play session. Finally, the mother completed any remaining surveys or forms.

### Coding

The films of the child's gaze during the IPL task were captured and digitized in the lab. Looking times were coded offline by watching these films frame by frame, using a custom coding program. The test audio was removed, so the coders did not know which direction of looking was correct. Looking during each frame was coded as to the left, right, center, or away. If a child did not look at both screens for more than 1 s total for a given trial, his/her data were not included for that trial. For the wh-question video, this occurred in 1.4% of test and control trials for the TD group and 4.6% of test and control trials for the ASD group. For the word order video, the percent of excluded trials for the TD group was 2.7%, and it was 2.9% for the ASD group. This level of data loss is similar to that in other IPL studies (Naigles et al., 2005; Swensen et al., 2007; Goodwin et al., 2012). All participants were coded by at least two coders to ensure reliability. The correlation between coders averaged 0.99, *p* < 0.001.

#### Wh-question comprehension

The dependent variable was the mean proportion of time that the child looked at the named item during each trial type (i.e., subject-, object-, and where-questions). This was the metric employed by Seidl et al. (2003; see also Goodwin et al., 2012) to demonstrate what-question comprehension; namely, the child needed to look at the named item significantly less during a subject- or object-wh-question trial than during the where-question trial. For example, to assess comprehension of “What tickled the bird?”, we compared children's looking time to the bird during this trial vs. during the “Where is the bird?” trial. During the “where” trial, they should look consistently at the bird whereas during the “what” trial, they should look consistently away from the bird. Such within-subject comparisons are common with the IPL paradigm, as children's eye movements during baseline trials serve as their own controls for performance during test trials (Brandone et al., 2007; Swingley, 2011; Piotroski and Naigles, 2012). To succeed at this task, then, children need not manifest a completely adult-like understanding of the grammar; they need only to allow the “what” questions to pull their attention away from the named item, indicating that they are aware that grammatical wh-movement has occurred (and that for object questions, SVO is no longer the correct word order). There is evidence that adults, too, initially look at the named item before switching to the correct referent, during online processing of what-questions (Sussman and Sedivy, 2003; Kukona and Tabor, 2011).

#### Word-order comprehension

The dependent variable was the difference score between the children's proportion of looking to the match during the test trial and baseline trials. This is a common way to assess comprehension via IPL s (Piotroski and Naigles, 2012); the test-baseline comparison demonstrates the degree to which the test audio guided the children's looking at the matching scene, relative to their initial preference for that scene based solely on stimulus salience. Data from visits 1 and 2 were combined (as in Tovar et al., 2015).

### Data analysis plan

In our first set of analyses, we assessed wh-question comprehension via repeated-measures ANOVAs to compare children's percentage of looking at the named item for the “where” question to looking at the named item for the “what” questions in each group. Next, we conducted pairwise correlations between the wh-question comprehension measures (using the difference score of percent looking to the named item during “where” questions minus percent looking to the named item during “what” questions) and standardized test language measures to discover relationships between children's general language and their wh-question comprehension. Finally, we conducted regression analyses to investigate the extent to which the children's performance on the earlier word order IPL measure (i.e., the grammatical measure) and their earlier Vineland communication and socialization scores (i.e., the social-pragmatic measures) uniquely predicted their performance on the later wh-question comprehension measure. These Vineland scores were entered separately as well as an average score.

## Results

### When do children with ASD and TD children comprehend wh-questions?

A repeated-measures analysis of variance (2 × 4 × 2) was conducted with group (ASD or TD) as the between-subjects variable, and visit (3, 4, 5, or 6) and trial type (combined subject- and object what questions, and where questions) as within-subjects variables. The results showed a main effect of trial [*F*_(1, 24)_ = 45.97, *p* < 0.001, partial eta squared = 0.657], indicating that children's proportion of looking to the named object was different for the “what” and “where” questions. There was no main effect of visit [*F*_(3, 72)_ = 0.988, *p* = 0.404, partial eta squared = 0.040], nor a significant group × trial interaction [*F*_(1, 24)_ = 1.15, *p* = 0.294, partial eta squared = 0.046]. A significant group effect emerged [*F*_(1, 24)_ = 8.92, *p* = 0.006, partial eta squared = 0.271], with greater overall looking to the named object by the TD group than by the ASD group. Given these significant trial and group effects, the next set of analyses investigated each group's looking patterns separately for subject- and object-what questions.

For the TD group, the first repeated-measures analysis of variance (4 × 2) was conducted with visits (3, 4, 5, 6) and trial type (*subject*-what questions and where questions) as within-subject variables. There was a main effect of trial [*F*_(1, 15)_ = 45.31, *p* < 0.001, partial eta squared = 0.751] but no main effect of visit [*F*_(3, 45)_ = 0.328, *p* > 0.05, partial eta squared = 0.021]. The visit × trial interaction trended toward significance [*F*_(3, 45)_ = 2.56, *p* = 0.068, partial eta squared = 0.145]. The second ANOVA compared the *object* wh-questions and “where” questions, and revealed a main effect of trial [*F*_(1, 15)_ = 30.63, *p* < 0.001, partial eta squared = 0.671], and no main effect of visit [*F*_(3, 45)_= 1.13, *p* > 0.05, partial eta squared = 0.070]. This visit × trial interaction also trended toward significance [*F*_(3, 45)_ = 2.55, *p* = 0.068, partial eta squared = 0.145].

For the ASD group, the first repeated-measures analysis of variance (4 × 2), conducted by visit (3, 4, 5, 6) and trial type (*subject*-what questions and where questions), revealed a main effect of trial [*F*_(1, 9)_ = 6.24, *p* < 0.05, partial eta squared = 0.409] but no main effect of visit [*F*_(3, 27)_ = 1.13, *p* > 0.05, partial eta squared = 0.112] and no visit × trial interaction [*F*_(3, 27)_ = 0.224, *p* > 0.05, partial eta squared = 0.024]. Similarly, the repeated-measures analysis of variance (4 × 2) for the object wh-questions with visit (3, 4, 5, 6) and trial type (*object*-what questions and where questions) revealed a main effect of trial [*F*_(1, 9)_ = 24.24, *p* < 0.005, partial eta squared = 0.729] but no main effect of visit [*F*_(3, 27)_ = 0.743, *p* > 0.05, partial eta squared = 0.076] nor a significant visit × trial interaction [*F*_(3, 27)_ = 0.374, *p* > 0.05, partial eta squared = 0.040].

**Figure 1:**
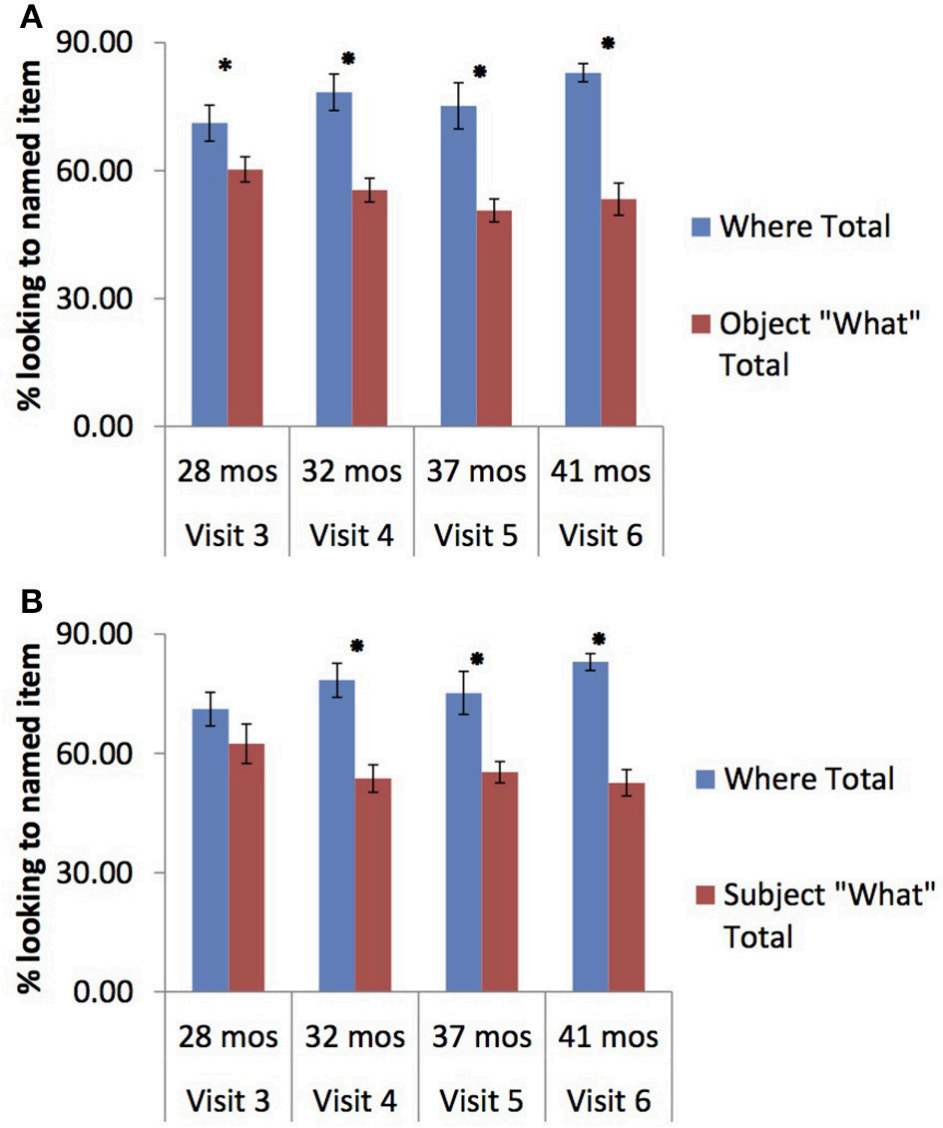
**(A)** Comparism of where vs. object trails for TD Children across visits. ^*^p < 0.05. **(B)** Comparism of where vs. subject trails for TD Children across visits. ^*^p < 0.05.

Overall, then, both groups demonstrated wh-question comprehension—they correctly looked *less* at the named item during the what-question trials than during the “where” trials. Because we were interested in when wh-question understanding was first achieved, and because of the marginal visit by trial interactions in the TD group, we next investigated each group's looking patterns for the subject and object wh-questions at each visit. For the purpose of these analyses, one-tailed significance testing was used as we expected an effect in a specific direction, i.e., *less* looking to the named item during the what-test trials. In the TD group, children looked significantly less to the named item during the object-what-trials vs. where-trials at all visits [visit 3: *t*_(16)_ = 1.90, *p* = 0.038; visit 4: *t*_(16)_ = 3.68, *p* = 0.001; visit 5: *t*_(16)_ = 4.09, *p* < 0.001; visit 6: *t*_(15)_ = 6.26, *p* < 0.001; see Figure 1A]; they also looked significantly less at the named item during subject what-questions compared to where-questions starting at visit 4 [visit 3: *t*_(16)_ = 1.27, *p* = 0.111; visit 4: *t*_(16)_ = 3.75, *p* < 0.001; visit 5: *t*_(16)_ = 3.57, *p* = 0.001; visit 6: *t*_(15)_ = 8.52, *p* < 0.001; see Figure 1B].

**Figure 2:**
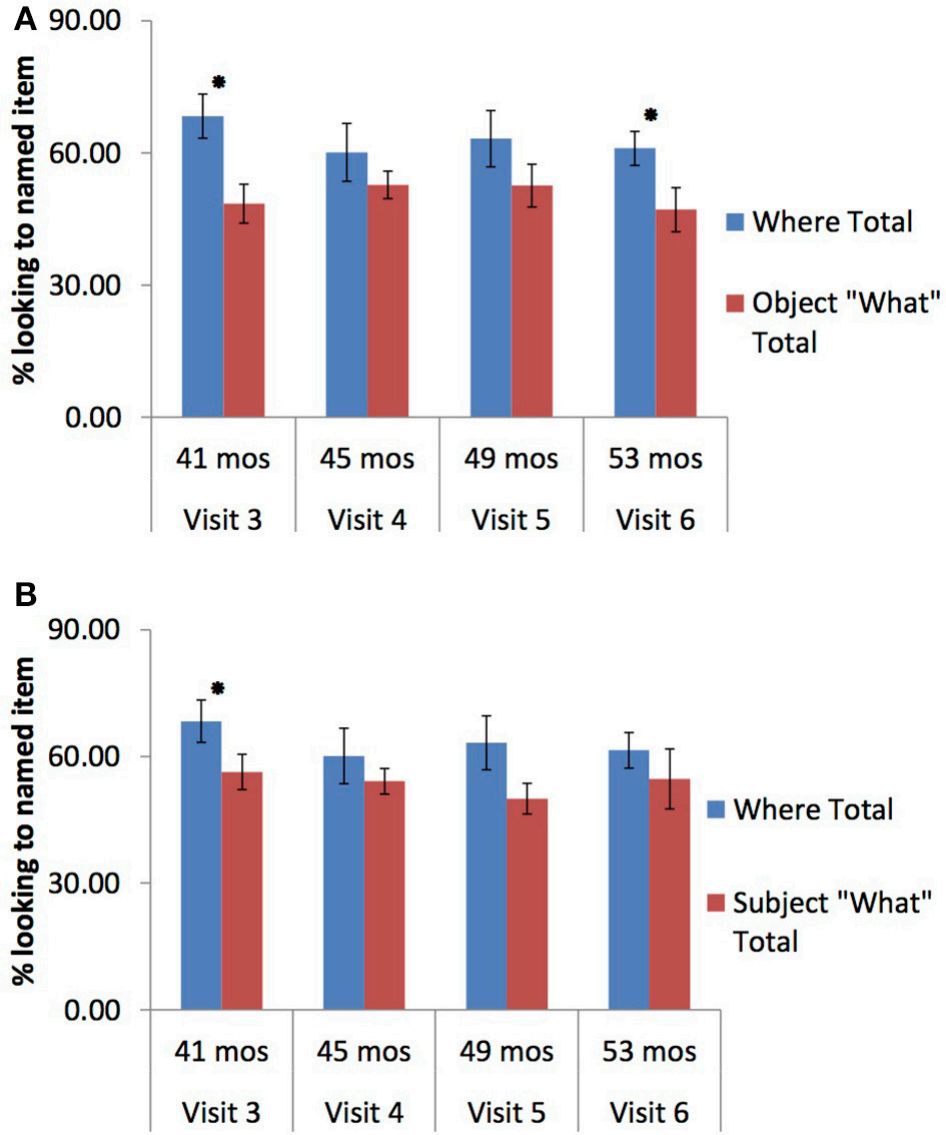
**(A)** Comparism of where vs. object what trails for Children with ASD across visits. ^*^p < 0.05. **(B)** Comparism of where vs. subject what trails for Children with ASD across visits. ^*^p < 0.05.

The ASD group's performance was less consistent for object-what questions: while they appeared to show comprehension at visit 3, *t*_(13)_ = 3.39, *p* = 0.002, this effect disappeared at visit 4, *t*_(13)_= 0.998, *p* = 0.168 and visit 5, *t*_(11)_ = 1.05, *p* = 0.157, then re-emerged at visit 6, *t*_(11)_ = 2.07, *p* = 0.031; see Figure 2A. Similarly, the ASD group's performance with subject-what questions varied across visits, reaching significance at visit 3 but then trending toward significance only at visit 5 [visit 3: *t*_(13)_ = 2.30, *p* = 0.019; visit 4: *t*_(13)_ = 0.807, *p* = 0.217; visit 5: *t*_(11)_ = 1.58, *p* = 0.07; visit 6: *t*_(10)_ = 0.857, *p* = 0.206; see Figure 2B].

In sum, TD children displayed evidence of wh-question comprehension by 32 months of age (i.e., visit 4, if both subject and object questions are considered). The ASD group demonstrated significant comprehension at visit 3; however, the ASD group was unable to maintain this level of comprehension consistently for the rest of the visits (with re-emerging significant comprehension for object wh-questions at visit 6). When the two groups are compared by age and/or visit, there is a discrepancy in the point of wh-question comprehension attainment; however, it is important to compare the groups by language level as well. As Table 1 shows, the two groups performed at equivalent language levels at visit 1, but by visit 3 they had diverged and the TD children were more advanced. We thus compared the language levels of the TD children at visit 4 and the children with ASD at visit 6; this comparison yielded no significant differences in receptive (ROWPVT) vocabulary [TD_visit4_: *M* = 43.31, *SD* = 11.95; ASD_visit6_: *M* = 48.28, *SD* = 19.35; *t*_(28)_ = −0.859, *p* > 0.05] or their expressive (EOWPVT) vocabulary [TD_visit4_: *M* = 31.17, *SD* = 9.89; ASD_visit6_: *M* = 30.00, *SD* = 24.55; *t*_(29)_ = −168, *p* > 0.05] Thus, it appears that the TD and ASD groups achieved comprehension of wh-questions at similar language levels.

We next consider the number of children in both groups at each visit who demonstrated wh-question comprehension. Difference scores were created for percent looking to the named item during “where” questions minus the same measure (combined across subject and object trials) during “what” questions. Positive scores indicated better understanding of wh-questions because these indicate that children looked longer at the named item during the “where” questions compared to the “what” questions; these children were designated “Comprehenders.” All children who showed a difference in the wrong direction (i.e., less than zero) were designated “Non-comprehenders.” A series of chi-square test of goodness-of-fit analyses {visit 3: [χ^2^_(1_, _*n*= 17)_ = 3.76, *p* = 0.05]; visit 4: [χ^2^_(1_, _*n*= 17)_ = 5.88, *p* < 0.05], visit 5: [χ^2^_(1_, _*n*= 17)_ = 8.48, *p* < 0.005]; and visit 6: [χ^2^_(1_, _*n*= 16)_ = 14.06, *p* < 0.001]}, indicate that in all the visits there were more Comprehenders than Non-comprehenders in the TD group. Within the ASD group, there were more Comprehenders than Non-comprehenders at visit 3 [χ^2^_(1_, _*n*= 14)_ = 5.78, *p* < 0.05; see Table 4].

**Table 4:** **Number of children showing comprehension or no comprehension of Wh-questions (subject—and object—questions combined)**.

**Visit**	**Comprehension type**	**TD**	**ASD**
Visit 3	Strong	13	12
	None	4	2
Visit 4	Strong	14	8
	None	3	6
Visit 5	Strong	15	7
	None	2	7
Visit 6	Strong	16	7
	None	0	7

*ASD, autism spectrum disorder; TD, typically developing*.

To further investigate individual differences, Pearson's correlations were conducted between measures of early language measures and concurrent or later wh-question comprehension scores (i.e., the difference scores). The five sets of language measures included the Vineland, Mullen, CDI, ROWPVT (receptive vocabulary) and EOWPVT (expressive vocabulary); a Bonferroni correction adjusted alpha to *p* = 0.005 was used as the threshold of statistical significance. As Table 5 shows, in the TD group, children with higher wh-question comprehension scores at visit 6 had had larger vocabulary scores (CDI) at visits 2 and 3 (*r*_*s*_ > 0.700, *p*_*s*_ < 0.005). Children with greater expressive vocabulary (EOWPVT) at visits 5 and 6 also had higher wh-comprehension scores at visit 6 (*r*_*s*_ > 0.700, *p* < 0.005; see Table 5). Due to the stricter significance level (*p* = 0.005), correlations among language measures and wh-question comprehension scores in the ASD group did not reach significance.

**Table 5:** **Cross-lagged and concurrent pearson correlations between language measures and Wh-question comprehension for TD children across all visits (***N*** = 17)**.

	**Visit 3**	**Visit 4**	**Visit 5**	**Visit 6**
**Variable**	**Wh-Q difference score**	**Wh-Q difference score**	**Wh-Q difference score**	**Wh-Q difference score**
**VISIT 1**				
MSEL	0.238	0.294	0.074	0.425
VABS	0.070	−0.358	−0.162	0.343
**VISIT 2**				
VABS	−0.242	−0.444	0.128	0.369
CDI	0.340	0.478	−0.148	0.714^*^
**VISIT 3**				
VABS	−0.071	−0.111	0.202	0.641^+^
CDI	0.077	0.302	−0.153	0.858^*^
**VISIT 4**				
VABS		−0.456	0.145	0.460
ROWPVT		0.451	0.182	0.579
EOWPVT		0.534	0.131	0.592
**VISIT 5**				
VABS			0.229	0.541
ROWPVT			0.177	0.504
EOWPVT			0.102	0.733^*^
**VISIT 6**				
MSEL				0.554
VABS				0.380
ROWPVT				0.639^+^
EOWPVT				0.780^*^

*MSEL, Mullen Scales of Early Learning Composite; VABS, Vineland Adaptive Behavior Scales Composite; CDI, Communicative Development Inventories; ROWPVT, Receptive One-Word Picture Vocabulary Test; EOWPVT, Expressive One-Word Picture Vocabulary Test*.

* p < 0.005, two-tailed;

+ *p < 0.01*.

### Do children's early comprehension of SVO word order and social competence predict their later comprehension of wh-questions?

We next analyzed the degree to which children's early understanding of canonical SVO word order, and their social competence, each independently predicted later wh-question comprehension. This kind of analysis is potentially perilous because of the small number of participants in each group (*n* = 15); moreover, eight children in this wh-question dataset were excluded from these regressions because their word order data were missing (e.g., because they did not look long enough at the video). Therefore, we increased our power by creating a larger dataset, which combined our participants and those of Goodwin et al. (2012; we also used the word order data first reported in Naigles et al., 2011). Combining the datasets is not automatically justified, because while the participant selection and procedures were identical, both the wh-question videos and the word order videos differed to some extent. However, our justifications for combining the datasets were as follows: First, as shown in Table 6, the language levels of the TD children in both datasets were equivalent at visits 1 and 6, and the language levels of the children with ASD in both datasets were also equivalent at visits 1 and 6. Second, whereas the characters for the two word order videos were different (girl and boy vs. horse and bird), the layouts themselves were almost identical, involving two animate characters and the five common transitive verbs and actions *push, tickle, wash, hug, and ride*. Third, whereas the wh-question stimuli were different across the videos (i.e., including inanimate agents and patients engaged in hitting actions in Goodwin et al. (2012); vs. animate agents and patients engaged in five reversible actions in the current study), these layouts were also almost identical (i.e., transitive actions followed by wh-object questions, transitive actions followed by wh-subject questions, then the where-questions). Fourth, the pattern of findings from the wh-question videos was similar in both datasets, with the TD children in both groups displaying stable comprehension of wh-questions by 32 months of age, and the children with ASD, in both groups demonstrating comprehension by 53–54 months of age (Goodwin et al., 2012). We believe these to be sufficient reasons for combining the datasets; however, we acknowledge that predictors of wh-question acquisition might vary according to animacy of the arguments (Tyack and Ingram, 1977; Philip et al., 2001). We defer further consideration of this point to the discussion section; for now, we consider the goal of discovering such predictors to warrant this exploratory analysis. Thus, the combined dataset for the word order-wh-question comparison now included 35 participants in the TD group and 31 in the ASD group.

**Table 6:** **Comparison of TD and ASD participants from both cohorts at visits 1 and 6 on the MSEL and CDI**.

	**Goodwin et al. (2012)**	**Current study**	***t***	***p*****-values**
	***M (SD)***	***M (SD)***		
**TD**				
**Visit 1**				
MLU	1.03 (0.04)	1.36 (0.25)	−5.38	<0.001
CDI	118.78 (114.35)	123.59 (108.15)	−0.128	0.899
Mullen receptive raw score	25.33 (2.93)	22.76 (3.87)	2.22^*^	0.033
Mullen expressive raw score	19.44 (4.46)	20.35 (5.70)	−0.527	0.602
**Visit 6**				
MLU	3.10 (0.43)	2.76 (0.54)	2.04	0.049
Mullen receptive raw	38.67 (4.13)	40.31 (4.88)	−1.07	0.295
Mullen expressive raw	39.72 (5.49)	39.69 (5.44)	0.018	0.985
**ASD**				
**Visit 1**				
MLU	1.04 (0.07)	1.26 (0.68)	−1.18	0.257
CDI	94.12 (111.38)	66.21 (113.60)	0.688	0.497
Mullen receptive raw	23.18 (8.19)	19.64 (10.37)	1.06	0.298
Mullen expressive raw	18.53 (8.13)	16.29 (6.64)	0.829	0.414
**Visit 6**				
MLU	2.01 (1.09)	1.97 (0.90)	0.106	0.915
Mullen receptive raw	31.18 (10.78)	34.21 (9.35)	−0.828	0.414
Mullen expressive raw	27.06 (13.31)	29.57 (13.78)	−0.515	0.611

MSEL, Mullen Scales of Early Learning. CDI, Communication Development Inventory;

* *p < 0.05*.

We conducted bivariate correlations between the word order measure, Vineland socialization, and communication scores separately and averaged, and subject and object wh-question comprehension scores at relevant visit. In the TD group, subject-wh-question comprehension at visit 5 was positively correlated with early word order comprehension (*r* = 0.359, *p* < 0.05) while subject wh-question comprehension at visit 6 was positively correlated with the averaged Vineland communication and socialization scores at visits 1 and 2 (*r* = 0.373, *p* < 0.05). In addition, object wh-question comprehension at visit 5 was positively correlated with visit 2 Vineland communication scores (*r* = 0.352, *p* < 0.05) while object wh-question comprehension at visit 6 was positively correlated with visit 1 and visit 2 Vineland communication scores (*r* = 0.370, *p* < 0.05; *r* = 0.373, *p* < 0.05) as well as the averaged Vineland communication and socialization score (*r* = 0.372, *p* < 0.05).

In the ASD group, visit 3 subject-wh question comprehension was significantly correlated with visit 2 Vineland communication (*r* = 0.438, *p* < 0.05) and the averaged Vineland socialization and communication score (*r* = 0.394, *p* < 0.05); furthermore, object wh-question comprehension at visit 6 was positively correlated with early word order comprehension (*r* = 0.381, *p* < 0.05).

We then conducted two stepwise multiple regressions, with each group separately, to assess the degree to which early word order understanding and early social/pragmatic performance uniquely contributed to later wh-question comprehension. Thus, the models included the children's word order scores, their visit 1 Mullen visual reception scores, their visit 2 CDI (language) scores, their visit 1 and visit 2 Vineland communication scores, and the average of the Vineland communication and socialization score. A measure of visual reception was included because this taps into children's non-verbal IQ, which is an important indicator of the children's ability to attend to and learn from their world. CDI scores from visit 2 were included to examine how an early vocabulary measure contributed to their later language processing ability, and the word order and Vineland communication and combined communication/socialization scores were early indicators of the children's grammatical and pragmatic abilities, respectively

In the TD group, the first regression model used visit 5 object-wh-question comprehension score as the outcome variable, yielding a significant model in which visit 2 communication scores were the only significant predictor *F*_(1, 30)_ = 4.97, *p* = 0.033 (see Table 7). The second regression model used visit 6 object-wh question comprehension score as the outcome variable, yielding a significant model in which visit 1 communication scores were the only significant predictor *F*_(1, 30)_ = 6.94, *p* = 0.013 (see Table 8). The third regression model used visit 6 subject wh-question comprehension score as the outcome variable, yielding two significant models. In the first model, the average of the Vineland communication and socialization scores was the significant predictor *F*_(1, 30)_ = 5.57, *p* = 0.025, whereas in the second model, both the average of the Vineland communication and socialization scores plus the word order scores each contributed significantly to the model, *F*_(2, 29)_ = 5.66, *p* = 0.008 (see Table 9).

**Table 7:** **Stepwise regression analysis for variables predicting visit 5 object—what question comprehension in TD children (***N*** = 31)**.

**Variable**	**B**	**SE(B)**	**β**	***t***	***p***	***R***^2^
Model 1					0.033	0.142
V2 communication	0.853	0.383	0.377	2.23	0.033	

*V2, Visit 2*.

**Table 8:** **Stepwise regression analysis for variables predicting visit 6 object—what question comprehension in TD children (***N*** = 33)**.

**Variable**	**B**	**SE(B)**	**β**	***t***	***p***	***R***^2^
Model 1					0.013	0.188
V1 Communication	1.03	0.391	0.433	2.64	0.013	

*V1, Visit 1*.

**Table 9:** **Stepwise regression analysis for variables predicting visit 6 subject—what question comprehension in TD children (***N*** = 33)**.

**Variable**	**B**	**SE(B)**	**β**	***t***	***p***	***R***^2^
Model 1					0.025	0.157
Vineland average	0.916	0.388	0.396	2.36	0.025	
Model 2					0.008	0.281
Vineland average	1.184	0.384	0.511	3.084	0.004	
Word order	51.88	23.20	0.371	2.24	0.033	

*Vineland composite, average of vineland socialization, and communication scores at visit 1 and 2*.

In the ASD group, the first regression model used visit 6 object-wh question comprehension as the outcome variable, yielding a significant model in which children's word order scores was the only significant predictor *F*_(1, 27)_ = 4.40, *p* = 0.045 (see Table 10). The second regression model used visit 3 subject-wh-question comprehension as the outcome variable, yielding a significant model in which visit 2 Vineland communication scores was the only significant predictor *F*_(1, 25)_ = 6.86, *p* = 0.015 (see Table 11).

**Table 10:** **Stepwise regression analysis for variables predicting visit 6 object—what question comprehension in children with ASD (***N*** = 29)**.

**Variable**	**B**	**SE(B)**	**β**	***t***	***P***	***R***^2^
Model 1					0.045	0.140
Word order	75.94	36.20	0.374	2.10	0.045	

**Table 11:** **Stepwise regression analysis for variables predicting visit 3 subject—what question comprehension in ASD children (***N*** = 27)**.

**Variable**	**B**	**SE(B)**	**β**	***t***	***p***	***R***^2^
Model 1					0.015	0.215
V2 Communication	0.843	0.322	0.464	2.62	0.015	

*V2, Visit 2*.

## Discussion

In this study, we addressed two main questions: (a) Viewing these new wh-question videos, which included animate agents and familiar actions and verbs, did children with ASD demonstrate comprehension of subject- and object-wh-questions at the same visit or language level as the TD children? (b) Did children's earlier grammatical knowledge (indexed by comprehension of SVO word order) and their social competence (indexed by their Vineland communication and socialization scores) predict their later comprehension of wh-questions? Addressing our first question, with these new videos, we found overall significant comprehension of wh-questions by both groups (i.e., a main effect of trial, with the children understanding that “where” questions asked them to look at the named item whereas subject and object “what” questions asked them to look away from the named item). More detailed scrutiny of performance at each visit, though revealed that TD children demonstrated robust comprehension of both subject- and object-questions by 32 months of age (i.e., at visit 4) whereas children with ASD showed what looked like comprehension at visit 3, which disappeared for visits 4 and 5 and then re-emerged at visit 6 (i.e., at 53 months of age), most strongly for the object wh-questions. Because their performance was not consistent across the first three visits when they viewed the wh-question video, we are cautious about claiming wh-question comprehension in the ASD group before visit 6. The two groups thus achieved wh-question comprehension at different ages and visits; however, the language level of the ASD group at visit 6, when they showed comprehension of object-wh-questions, was quite similar to those of TD children at visit 4, the earliest visit when these children showed stable comprehension of both object-wh and subject-wh-questions.

Addressing our second question, we found that wh-question comprehension was related to both grammatical and social-communication abilities. That is, for both TD children and children with ASD, their comprehension of SVO word order as well as their Vineland social-pragmatic scores at earlier visits predicted their later performance on wh-question comprehension.

Our new wh-question videos were designed with the goal of making wh-question processing easier, because we included animate subjects—who are the typical agents in prototypical transitive actions—and verbs that were more familiar to both TD children and children with ASD. Therefore, we expected to find robust subject- and object- wh-comprehension performance in our TD group at visit 3 (the first time they saw the video), replicating Goodwin et al. (2012), and earlier subject and object wh-question comprehension in the ASD group than had been found by Goodwin et al. (2012). However, our results were, somewhat surprisingly, quite parallel to those of Goodwin et al. (2012), with the TD group showing marginal comprehension at visit 3 and robust comprehension at visit 4, and the ASD group still showing inconsistent comprehension across visits. Thus, the new videos did not elicit earlier evidence of comprehension from the ASD group. Replicating Goodwin et al. (2012), we found that the groups appeared to achieve good “what” question comprehension when their language levels were on par; that is, at visit 4 for the TD group and visit 6 for the ASD group. Interestingly, though, we did not replicate the correlations that Goodwin et al. (2012) observed, in the ASD group, between vocabulary levels and wh-question comprehension; possibly, this discrepancy indicates that the children who achieved good wh-question performance with the Goodwin et al. (2012) video were the ones who knew the verb “hit,” whereas no such association was observed with the current videos because all verbs were familiar. Taken together, these findings suggest that using familiar verbs and animate agents did not change the basic findings of Goodwin et al. (2012); namely, that wh-questions are difficult for children with ASD. Even though children were only required to *look* at the correct answer, they still demonstrated impairments in their understanding. We suggest that these findings support the argument that these children's difficulties with wh-questions have a grammatical-origin.

We also investigated the degree to which children's variance in their early grammatical and/or social-pragmatic performance might predict their later variance in subject and object-wh question comprehension. Indeed, the regressions suggested that wh-question comprehension is related to both grammatical and social-pragmatic factors. The “grammatical-origins” argument is supported because the children's performance on the earlier word order task strongly predicted performance on later wh-question comprehension, for both the TD and ASD groups (albeit at different visits and for different wh-questions). These relationships held even when non-verbal cognition and general vocabulary level were controlled; therefore, they are not indicators of general ability to perform well in cognitively or linguistically demanding tasks. We suggest, instead, that the children's competence at understanding the canonical English SVO word order helped them become more efficient in subsequently processing wh-questions, in that having stable representations of SVO helped them understand that the moved wh-word in a subject-wh or object-wh-question maps onto the grammatical subject or object of the verb, respectively. These findings provide evidence for the continuity of grammatical knowledge in both young TD children and children with ASD, such that they might use early-developing syntactic knowledge to process the grammatical role of wh-words.

These findings extend those of Naigles et al. (2011), who demonstrated that children with ASD who were faster at understanding SVO sentences were also better at using such transitive frames to conjecture that the novel verbs in them were causative; i.e., doing syntactic bootstrapping. That correspondence was thus between understanding SVO sentences with familiar verbs and learning verbs in SVO sentences with novel verbs—i.e., both tasks involved essentially the same sentence forms. Our current findings extend Naigles et al. (2011) because we have demonstrated correspondences between understanding canonical SVO frames at early visits and understanding *non*-canonical SVO frames at later visits. That is, the children in the current study needed to understand that the fronted wh-word “stood for” an NP, and to know that the NP-trace was either in subject or object position. Moreover, when the NP-trace was in object position, the surface word order was OVS; thus, the correspondence we observed in the ASD group between SVO comprehension at visits 1–2 and object-wh-question at visit 6 suggests that the children with ASD are not perseverating on one specific word order and had some knowledge of the abstract relationship between sentences that had different surface orders. This observed correspondence thus supports the argument that the wh-question deficit in children with ASD has a grammatical origin.

However, our findings also support the argument that wh-question impairments in children with ASD also derive from pragmatic impairments. That is, the TD group and the ASD group's comprehension of wh-question at the later visits was predicted by their social-pragmatic abilities at the earlier visits, in that children with better performance on wh-question comprehension were reported by their parents to have better communication and socialization skills on the Vineland. Social-pragmatic abilities might play a role in the development of wh-question understanding in both general and specific ways. In general terms, children who are more attuned to their social environment might simply pay more attention to the language their parents use, which would include wh-questions (see also Goodwin et al., 2015). In specific terms, children who are more aware of the social conventions about when and how to ask wh-questions, and who pay attention to their parents' pointing to objects when they (the parents) ask questions, would be expected to better understand the referents of wh-questions. When children are more attuned to their social environment, they can better understand the focus and interpretation of how questions are used and formulated by their family members. Better social-pragmatic abilities would enable children to understand the different functions of wh-questions and the particular context within which they are used which can strengthen their knowledge and understanding of wh-questions.

Limitations of this study include participant characteristics, our choice of social-pragmatic measures and a lack of a joint attention measure, and the wh-question video itself. First, we are restricted in the generalizability of these findings with children with ASD as these children were receiving ABA as their primary intervention, and therefore the generalizability of these findings to the ASD population as a whole are limited. Second, we are limited in our argument to further distinguish syntactic challenges from pragmatic challenges, as this study did not analyze children's production data of wh-questions or their joint attention skills; that is, we are limited in our knowledge about whether children in our study also showed deficits in their wh-question production, indicating a pragmatic challenge (however, note that Goodwin et al. (2012) found delays in both production and comprehension of wh-questions). Also, joint attention would be a key predictor to investigate in future studies because it taps into pragmatic skills in children and therefore it would be important to examine whether joint attention skills are related to later syntactic development. Perhaps, if their joint attention is impaired, then we might also see pragmatic aspects of their wh-question production being impaired. Third, it is possible that we made the wh-question task harder for children with ASD by using two animate characters engaged in causative actions. As has been shown in prior research, a prototypical action is an animate object performing an action on an inanimate object (Slobin, 1982). Perhaps our inclusion of animate patients in the current wh-question video made wh-question processing more challenging, possibly even for both groups (but see Gagliardi et al., 2016, who found good wh-question comprehension in TD toddlers who viewed videos with animate patients). In line with this, another limitation is that we combined the wh-question video with animate characters with the wh-question video with inanimate characters in our prediction analyses and it is possible that there can be different predictors for animate characters and inanimate characters. For example, Tyack and Ingram (1977) and Philip et al. (2001) found that typical children's acquisition of “who” and “what” questions emerged at different ages. It is important to point out that our study controlled for that by asking “what” questions throughout. It is possible that TD children in our study did not show early stable comprehension of wh-questions as their peers did in Goodwin et al. (2012) because we used animate characters with “what” questions. We believe that this would not be an issue for children with ASD because of their pragmatic impairment; however, this remains to be an open question.

In future work, it would be interesting to discover extent of the impairment in wh-questions in other languages, and investigate whether the deficits in understanding such wh-questions also hold for languages that do not require wh-movement. Members of our group have used Goodwin et al.'s (2012) video to examine wh-question comprehension in South Korean children with ASD, with the preliminary finding that, even though Korean wh-words remain in situ, Korean 4-year-olds with ASD nonetheless show poorer wh-question comprehension than their language-matched TD peers (Park et al., 2016). This is an important step toward determining which grammatical components of wh-questions are most challenging for children with ASD. Additionally, we concluded that the children with ASD showed comprehension at visit 6 rather than at visit 3 because they did not show comprehension at visits 4 and 5; however, this U-shaped curve is puzzling and future studies are needed to replicate this effect.

In conclusion, the IPL paradigm has elicited comprehension of wh-questions in 2-year-old TD children; in contrast, children with ASD demonstrated delayed and somewhat inconsistent understanding of these same wh-questions. Changing the actions to more familiar ones did not help children with ASD demonstrate earlier comprehension compared to previous results (Goodwin et al., 2012). Our findings suggest that wh-questions present linguistic challenges to children with ASD that go beyond issues of stimuli. They lend support to both “grammatical-origins” and “pragmatic-origins” hypotheses concerning the wh-question deficit in children with ASD: The “grammatical-origins” argument is supported because performance on an early grammatical competence task was strongly associated with performance on later wh-question comprehension for both groups. The “pragmatic-origins” argument is also supported because wh-question comprehension was associated with children's earlier social-communication scores, i.e., children with better social abilities were later more able to consistently comprehend wh-questions. Thus, the current study shows that wh-question challenges seem to be related to both grammatical and pragmatic challenges in children with ASD.

Finally, our finding that both linguistic and social-pragmatic factors are implicated in wh-question acquisition in children with ASD is consistent with the recent report of Naigles et al. (2016), who found that children with ASD's vocabulary and joint attention skills each independently predicted their propensity to reverse personal pronouns. These studies provide the first demonstrations that both specifically linguistic and generally social factors are influential in the language challenges of children with ASD, and we encourage more researchers to include measures that tap into multiple domains when they are investigating the language of these individuals. We suggest that attributing the language challenges of children with ASD to “only” linguistic or social bases masks the intricate coordination that children perform—even children with ASD—among multiple domains of knowledge during language development.

## Ethics statement

This study was carried out in accordance with the recommendations of the University of Connecticut, Institutional Review Board with written informed consent from all subjects. All subjects gave written informed consent in accordance with the Declaration of Helsinki. The protocol was approved by the UConn IRB.

## Author contributions

LN and DF designed the original data collection. MJ and LN worked together on the questions, design, coding, analyses, and write up of the current study, with some input from DF.

## Funding

This research was funded by the National Institute on Deafness and Other Communication Disorders (NIH-DCD) [grant number R01 2DC007428].

### Conflict of interest statement

The authors declare that the research was conducted in the absence of any commercial or financial relationships that could be construed as a potential conflict of interest.
